# Association of Brain Metastases With Immune Checkpoint Inhibitors Efficacy in Advanced Lung Cancer: A Systematic Review and Meta-Analysis

**DOI:** 10.3389/fonc.2021.721760

**Published:** 2021-12-08

**Authors:** Yanning Wang, Qianning Zhang, Chuansheng Chen, Yuxuan Hu, Liyun Miao, Yujie Zhou

**Affiliations:** ^1^ Clinical Stem Cell Center, The Affiliated Drum Tower Hospital of Nanjing University Medical School, Nanjing, China; ^2^ School of Basic Medicine and Clinical Pharmacy, China Pharmaceutical University Nanjing Drum Tower Hospital, Nanjing, China; ^3^ Institute of Pharmaceutical Sciences, China Pharmaceutical University, Nanjing, China; ^4^ Department of Respiratory and Critical Care Medicine, The Affiliated Drum Tower Hospital of Nanjing University Medical School, Nanjing, China

**Keywords:** immune checkpoint inhibitors, survival, brain metastases, lung carcinoma, meta-analysis

## Abstract

**Background:**

In pivotal immunotherapy trials, the efficacy of immune checkpoint inhibitors as treatments for lung cancer patients with brain metastases remains controversial. The aim of this study was to assess the relative efficacy of immunotherapy versus standard systemic therapy in advanced lung cancer patients with and without brain metastases.

**Methods:**

Systematic searches of PubMed, Embase, Cochrane database, and conference proceedings up to Aug 6, 2020 without year and language restrictions. The main outcomes were the overall survival in patients with and without brain metastases measured by hazard ratios, and the difference in efficacy between patients with and without brain metastases was measured by ratio of hazard ratios.

**Results:**

Nine eligible randomized controlled trials involving 6241 patients (682 [11%] with brain metastases and 5559 [89%] without brain metastases) were included in the analysis. A survival benefit of immunotherapy was observed for both patients with brain metastases (HR, 0.75; 95%CI, 0.53-0.97; P = .026) and patients without brain metastases (HR, 0.75; 95%CI, 0.67-0.83; P <.001). However, patients without brain metastases benefit more from immunotherapy than patients with brain metastases (HR, 1.37; 95%CI, 1.15-1.63; P = .001). Additionally, subgroup analyses indicated that tumor type affect the efficacy of immunotherapy in patients with brain metastases (HR, 1.04 vs 1.54; interaction, P = .041).

**Conclusions:**

Immunotherapy can significantly improve overall survival for advanced lung cancer patients with asymptomatic brain metastases, especially in patients with non-small-cell lung cancer, but the magnitude of benefit is brain metastases dependent.

**Systematic Review Registration:**

https://www.crd.york.ac.uk/prospero/display_record.php?ID=CRD42020206597.

## Background

Brain is a common metastatic site of lung cancer. With the emergence of novel systemic treatments and the development of diagnostic imaging technology, survival time of cancer patients extended which increasing the chance of developing brain metastases, and the detection rate of brain metastases has increased (1). At diagnosis, approximately 10% of patients with non-small-cell lung cancer (NSCLC) have brain metastases, and 20%-40% will develop brain metastases during their illness (2). Occurrence of brain metastases is related to poor prognosis, with a median overall survival (OS) of only 1-2 months in untreated patients (3).

The main treatments available for patients with a limited number of brain metastases are surgery and stereotactic radiotherapy (4). Whole-brain radiotherapy is the standard of care for patients with symptomatic multiple brain metastases (5). The management of multiple asymptomatic brain metastases usually adopts systemic therapy (6). Chemotherapy is uncommonly used due to the limitation of crossing the blood-brain barrier (7), while new generation of anaplastic lymphoma kinase (ALK) or epidermal growth factor receptor (EGFR) tyrosine kinase inhibitors have shown activity in treating brain metastases (8, 9). However, systemic therapy for patients without sensitizing mutations are still lacking.

Despite dramatically changed the treatment landscape of advanced lung cancer with the emergence of immune checkpoint inhibitors (ICIs), there was still a degree of caution, about using these new drugs for the treatment of lung cancer patients with brain metastases. This hesitation can be explained by the inherent concept that brain has been considered an ‘immune-privileged’ organ (10). In the past, an absent lymphatic system and blood-brain barrier (BBB) were considered responsible for poor brain immunogenicity. However, since the discovery of an intact central nervous system lymphatic system, the traditional understanding of the brain microenvironment has been refuted (11). Studies have found that resident cells (microglia, macrophages, and CD4+memory T cells) in the brain, performing immune surveillance continuously, and once the intracranial tumorigenesis, the BBB becomes more permeable to promote immune cell infiltration (10, 12–14). In animal models, the immune crosstalk between brain tumors and extracranial tumor after using immune checkpoint blockade has been demonstrated (15). Thus, it has been speculated that ICIs may have a therapeutic effect on brain metastases (16).

Recently, in a phase II trial of pembrolizumab in patients with NSCLC and brain metastases, an encouraging 29.7% intracranial overall response rate (ORR) was observed in a selected population with programmed death ligand 1 expression at least 1%, which was similar to its systemic activity (17). However, outcomes of patients with brain metastases in pivotal immunotherapy trials vary, with sometimes a benefit of immunotherapy over standard of care, but sometimes not.

Currently, studies exploring the interaction between the efficacy of ICIs and brain metastases are scarce. Here, with recently accumulated evidence, to address this question, we performed this meta-analysis that examines the efficacy of immunotherapy in advanced lung cancer patients with and without brain metastases.

## Materials and Methods

### Search Strategy and Selection Criteria

This meta-analysis followed the PRISMA guidelines (18). The prospective protocol has been uploaded to the PROSPERO platform. We searched the Cochrane Library, PubMed, and Embase for phase 2 or 3 randomized controlled trials up to Aug 6, 2020 without year and language restrictions, We also searched conference proceedings from European Society of Medical Oncology and American Society of Clinical Oncology. Two investigators (Q.Z. and C.C.) independently performed literature searches. The search terms were ipilimumab, tremelimumab, pembrolizumab, atezolizumab, durvalumab, avelumab, cemiplimab, camrelizumab, sintilimab, tislelizumab, toripalimab, immune checkpoint inhibitor, CTLA-4, PD-1, PD-L1, and Lung Cancer (**Supplementary Table 1**). We also reviewed the references of reviews and the final included articles.

We included studies that assessed immunotherapy (ie, PD-1, PD-L1, and CTLA-4) in lung cancer, and had data available for OS according to the presence of brain metastases. We identified randomized controlled trials that compared ICIs alone or in combination with other drugs (chemotherapy or other ICIs), with placebo or non-ICIs. To prevent the inclusion of duplicate data, we selected the most recent publication from studies with the same patient cohort.

We excluded observational studies (ie, cohort and case-control studies), single-arm trials (ie, non-randomized trials), case report, case series, editorials, review articles. We sought to examine the difference in the efficacy of immunotherapy between lung cancer patients with and without brain metastases, studies that reported subgroup analysis for brain metastases (with or without) only were excluded.

Study selections were performed by two investigators (Q.Z. and C.C.) independently, and disagreements were resolved with the consensus of another two investigators (Y.W. and L.M.). We reviewed the full-text and supplemental content if titles and abstracts were insufficient for determining if studies met selection criteria.

### Data Extraction and Quality Assessment

Two investigators (Q.Z. and C.C.) independently developed a data extraction, and the third authors (Y.W.) conducted independent verification and resolved all discrepancies. Study characteristics, including trial name, first author and year of publication, study design and phase, study population, number of patients, mean age, line of therapy, intervention and comparison, median follow-up time, hazard ratio (HR) for death stratified by brain metastases (with and without). We used the Cochrane Collaboration tool to conduct the risk-of-bias assessment (19).

### Outcome Measures

The primary outcomes were OS measured with HR for death and 95% confidence intervals (CI) stratified by brain metastases, and the difference in efficacy between patients with brain metastases and without brain metastases measured in terms of the OS difference.

### Statistical Analysis

We derived the HR for death (with 95% CI) from each study, separately for patients with and without brain metastases. We used the inverse variance method to calculate the pooled HR of death. Considering the clinical heterogeneity of the data, the pooled HR was calculated by random-effects models. We did the Q-test and I² statistics to identify heterogeneity (20). To assess the difference of immunotherapy effect between lung cancer patients with and without brain metastases, we adopted the following approach: calculating an interaction HR (HR in patients with brain metastases/HR in patients without brain metastases) in each study, and then we pooled these interaction HRs using random-effects models (21).

We performed subgroup analyses to assess other factors in effect of brain metastases on immunotherapy efficacy. The subgroups were type of tumor (NSCLC, SCLC), type of therapy (ICIs alone, ICIs combination), line of therapy, target of intervention agents (anti-PD-L1, anti-CTLA-4, and anti-PD-1), and median follow-up duration. Publication bias was evaluated by the funnel plot.

We conducted all analyses using STATA (version 15.0). All reported p-values are two-sided, and p < 0.05 indicating statistical significance.

## Results

### Literature Search Results

**Figure 1:**
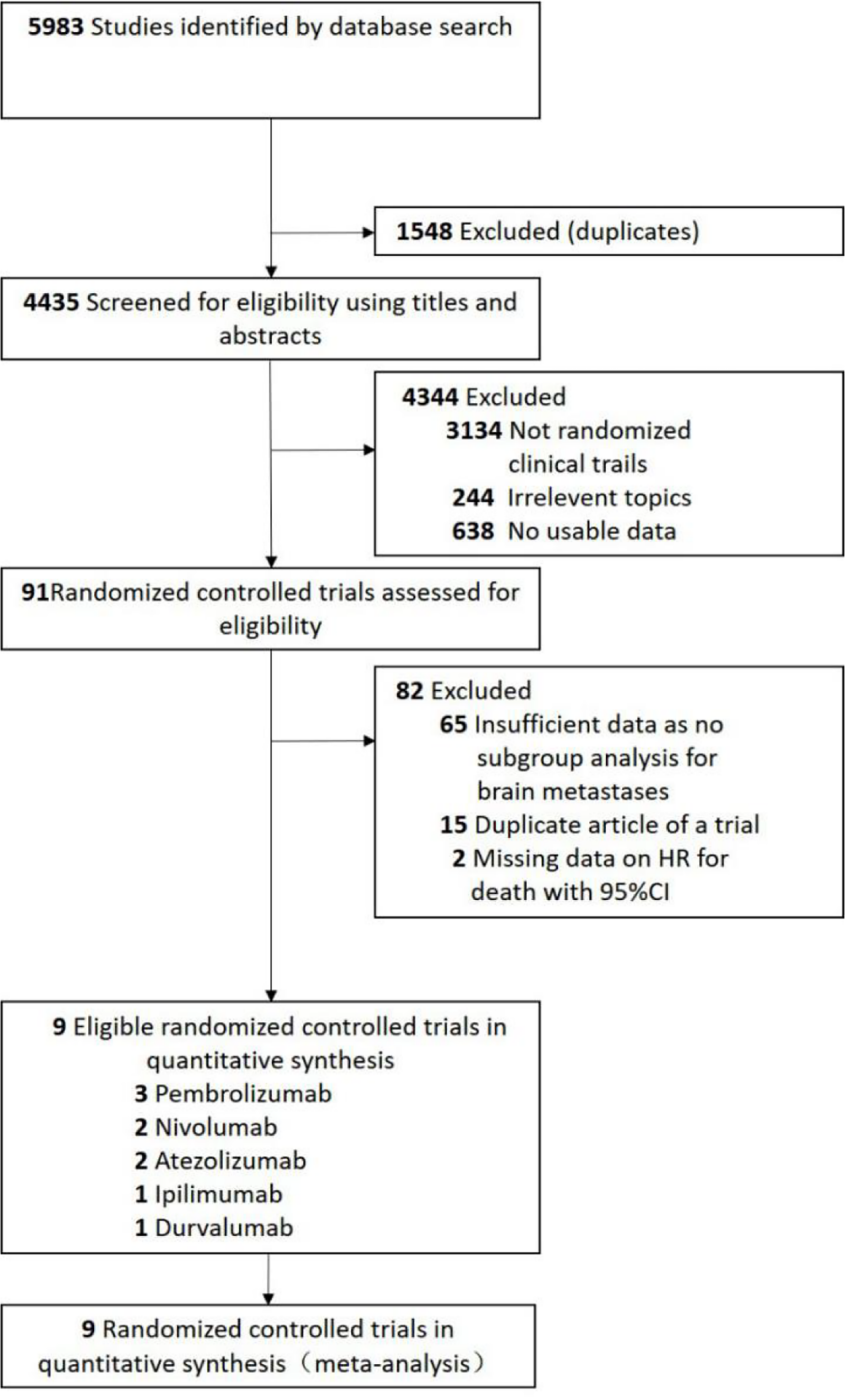
Flow Chart Diagram of Study Selection.

A total of 5983 studies were retrieved by initial search from the Cochrane Library, PubMed, and Embase. We removed 1548 studies because of duplications. Based on the review of the titles and abstracts, 4344 studies were excluded. After full texts review, 9 randomized controlled trials (22–30) were eligible for analysis (**Figure 1**). Four trials (22, 25, 28, 29) with PD-1 inhibitors (three with pembrolizumab and one with nivolumab), three trials (24, 26, 30) with PD-L1 inhibitors (two with atezolizumab and one with durvalumab), one trial (27) with anti-CTLA-4 antibodies (ipilimumab), and one trial (23) with ipilimumab and nivolumab in combination (**Figure 1**).

### Characteristics of Identified Trials

All the eligible trials (22–30) were international, multicenter phase 3 trials published between 2015 and 2020 (**Table 1**). Five trials (22, 23, 25, 26, 28) were done in patients with NSCLC, and four in patients with SCLC (24, 27, 29, 30). All trials were performed in advanced or extensive-stage settings. Seven trials (22–24, 26, 28–30) were performed in first-line settings and 2 in subsequent line settings (25, 27).

**Table 1:** Characteristics and outcomes of the 9 trials included in the meta-analysis.

Source(Trial Name)	Lines of Therapy	Tumour type	Phase	PD-L1 expression	Treatment groups	Patients	Brain metastases (No.)		Median age, years	Median follow-up (Range or IQR), mo	HRs for OS with Int vs Cont(95%CI)		
							YES	NO			Overall	With brain metastases	Without brain metastases
Gandhi et al,2018 (22)	1	nonsquamous NSCLC	3	any level	pembrolizumab+chemotherapy vs placebo+chemotherapy(2:1)	616	108	508	Int:65(34-84); Cont:63.5(34-84)	23.1(18.6-30.9)	0.56(0.45-0.70)	0.41(0.24-0.67)	0.59(0.46-0.75)
(KEYNOTE-189)													
Hellmann et al,2019 (23)	1	NSCLC	3	any level	nivolumab+ipilimumab vs chemotherap(1:1)	1166	115	1051	Int:64(26-87); Cont:64(29-87)	Minimum:29.3(NR)	0.73(0.64-0.84)	0.64(0.42-0.97)	0.75(0.65-0.86)
(CheckMate 227)													
Horn et al,2018 (24)	1	SCLC	3	NR	atezolizumab+chemotherapy vs placebo+chemotherapy(1:1)	403	35	368	Int:64(28-90); Cont:64(26-87)	13.9(NR)	0.70(0.54-0.91)	1.07(0.47-2.43)	0.68(0.52-0.89)
(IMpower133)													
Reck et al,2019 (25)	>1	NSCLC	3	≥50%	Pembrolizumab vs chemotherapy(1:1)	305	28	277	65(33-90)	25.2(20.4-33.7)	0.63(0.47-0.86)	0.73(0.20-2.62)	0.64(0.46-0.88)
(KEYNOTE-024)													
Fehrenbacher et al, 2018 (26) (OAK)	1	NSCLC	3	any level	atezolizumab vs docetaxel(1:1)	1225	118	1107	Int:63(25-84); Cont:64(34-85)	28(NR)	0.80(0.70-0.92)	0.59(0.38-0.92)	0.82(0.71-0.94)
Reck et al,2016 (27)	>1	SCLC	3	NR	ipilimumab+chemotherapy vs placebo+chemotherapy(1:1)	954	100	854	62(36-85)	10.5(NR) vs 10.2(NR)	0.94(0.81-1.09)	1.58(1.02-2.44)	1.03(0.88-1.20)
(CA184-156 study)													
Borghaei et al,2015 (28)	1	nonsquamous NSCLC	3	any level	nivolumab vs docetaxel(1:1)	582	68	514	62(21-85)	13.2(NR)	0.75(0.62-0.91)	1.04(0.62-1.76)	0.71(0.58-0.88)
(CheckMate 057)													
Rudin et al,2020 (29)	1	SCLC	3	any level	pembrolizumab+chemotherapy vs placebo+chemotherapy(1:1)	453	55	398	65(24-83)	21.6(range 16.1-30.6)	0.80(0.64-0.98)	1.32(0.72-2.42)	0.75(0.60-0.94)
(KEYNOTE-604)													
Paz-Ares et al,2019 (30)	1	SCLC	3	NR	durvalumab+chemotherapy vs chemotherapy(1:1)	537	55	482	63(57-68)	14.2(IQR 11.7-17.0)	0.73(0.59-0.91)	0.69(0.35-1.31)	0.74(0.59-0.93)
(CASPIAN)													

ALK, anaplastic lymphoma kinase; Cont, control group; EGFR, epidermal growth factor receptor; HR, hazard ratio; Int, intervention group; IQR, interquartile range; NR, not reported; NSCLC, non–small cell lung cancer; PD-L1, programmed cell death 1 ligand 1; SCLC, small cell lung cancer.

The number of patients enrolled in eligible studies ranged between 305 and 1225. Of the total 6241 patients included, 682 (11%) were patients with brain metastases and 5559 (89%) were patients without brain metastases. The median age ranged from 62 to 65 years, and median follow-up ranged from 10.2 to 25.2 months.

3673 (94%) of 3894 patients with NSCLC without sensitizing EGFR and ALK mutations. Two of five trials (26, 28) in patients with NSCLC enrolled any patients with EGFR or ALK mutations: in one trial, 113 (9%) of 1225 patients had EGFR mutation and 5 (<1%) had ALK mutation (26); in the other, 82 (14%) of 582 patients had EGFR mutation and 21 (4%) had ALK mutation (28). Most trials enrolled patients with any level of PD-L1 expression, one trial (25) only enrolled patients with PD-L1 expression on 50% or more.

The eligible criteria of included trials for patients with brain metastases were summarized in the **Supplementary Table 3** in the Supplement. Briefly, all of trials enrolled patients diagnosed with asymptomatic brain metastases prior to immunotherapy. Most trials excluded patients with ongoing steroids as therapy for brain metastases, only one trial (28) enrolled patients with stable or decreasing dose of prednisone (≤10mg daily).

### Risk of Bias

The risk of bias assessment is shown in the **Supplementary Table 2** in the Supplement. Several trials had a risk of performance bias due to open-label design. Most trials generated an appropriate allocation concealment. All trials had adequate randomization mode. All trials had a low risk of attrition bias and reporting bias.

### Primary Analysis

**Figure 2:**
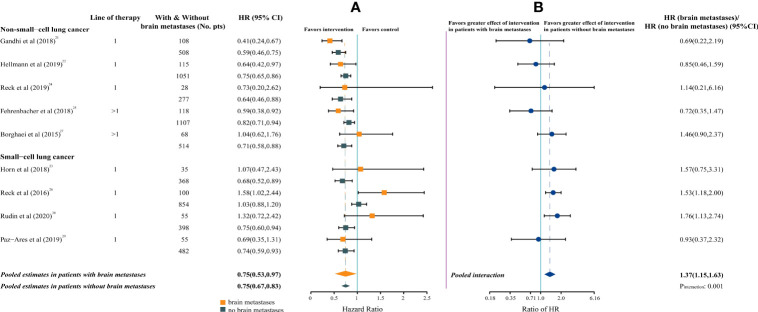
Forest Plot of Hazard Ratios for Death According to Brain Metastases. **(A)**, Hazard Ratios (HRs) of death for patients assigned to intervention group, compared with those assigned to control group, by brain metastases. **(B)**, The interaction between immunotherapy efficacy and brain metastases.

Compared with chemotherapy, a significantly survival benefit was observed for both patients with brain metastases (pooled overall survival HR, 0.75; 95%CI, 0.53-0.97; P = .026) and patients without brain metastases (HR, 0.75; 95%CI, 0.67-0.83; P <.001) treated with ICIs. Statistically significant inter-study heterogeneity was estimated among both patients with brain metastases (P = .037; I² = 51%) and patients without brain metastases (P = .011; I²= 59%) (**Figure 2A**).

The pooled ratio of HRs in patients with brain metastases versus patients without brain metastases was 1.37 (95%CI, 1.15-1.63; P = .001; I²= 16%), indicating a greater efficacy for ICIs in patients without brain metastases (**Figure 2B**).

### Subgroup Analysis

**Figure 3:**
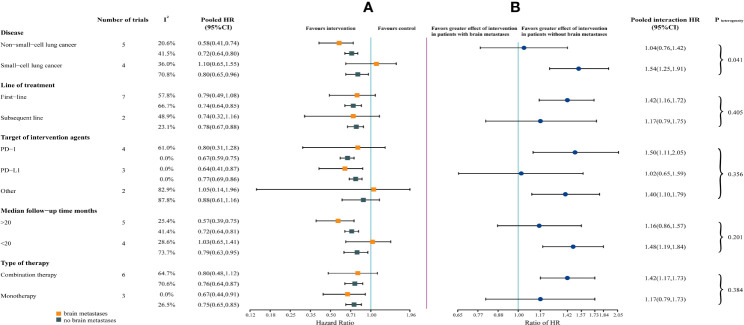
Forest Plot of Hazard Ratios for Death According to Brain Metastases, by Subgroup. **(A)** Hazard Ratios (HRs) of death for patients assigned to intervention group, compared with those assigned to control group, according to brain metastases and subgroups (ie, type of therapy, type of cancer, line of therapy, median follow-up duration, and target of intervention agents). **(B)** The interaction between immunotherapy efficacy and brain metastases, according to subgroups (ie, type of therapy, type of cancer, line of therapy, median follow-up duration, and target of intervention agents).

For each of the subgroups (ie, type of therapy, type of cancer, line of therapy, median follow-up duration, and target of intervention agents), patients without brain metastases could benefit from immunotherapy. However, patients with brain metastases could benefit from immunotherapy only in a subset of subgroups (ie, immune monotherapy, NSCLC, median follow-up > 20 months, and targeting PD-L1) (**Figure 3A**).

The survival benefit from ICIs was similar in patients with and without brain metastases in the following subgroups: immune monotherapy, NSCLC, subsequent line settings, median follow-up > 20 months, and targeting PD-L1. Notably, in non-small-cell lung cancer, the magnitude of efficacy of ICIs was greater for patients with brain metastases, compared with small-cell lung cancer (SCLC) (HR, 1.04 vs 1.54; interaction, P = .041) (**Figure 3B**).

### Sensitivity Analysis and Publication Bias

We performed two sensitivity analyses. In the first, we excluded the KEYNOTE-024 trial (25) that only enrolled patients with PD-L1 expression on 50% or more. In the second analysis, we excluded the CheckMate-057 trial (28) that enrolled patients with ongoing steroids as therapy for brain metastases. The pooled HRs reported in patients with brain metastases and pooled ratio of HRs were respectively 0.76 (95%CI, 0.53-0.99; P = .041; I²= 57%) and 1.37 (95%CI, 1.15-1.63; P = .001; I²= 26%) for the first sensitivity analysis (**Supplementary Figure 1**), 0.71 (95%CI, 0.49-0.94; P = .012; I²= 51%) and 1.35 (95%CI, 1.12-1.63; P = .002; I²= 26%) for the second (**Supplementary Figure 2**). These results were consistent with our findings.

No evidence of publication bias was detected by funnel plot (**Supplementary Figure 3**).

## Discussion

With published data from 9 multicenter phase 3 randomized controlled trials, for more than 6000 patients with advanced lung cancer, our pooled analysis showed that, compared with chemotherapy, ICIs can decrease the risk of death for both patients with and without brain metastases, especially in patients with NSCLC. However, patients without brain metastases have a larger treatment effect from ICIs versus chemotherapy than do patients with brain metastases.

To the best of our knowledge, there are no analysis that specifically explores the efficacy of ICIs in lung cancer patients with brain metastases, partly because only limited data on the survival of patients with brain metastases is available. The available evidence mainly generates from expanded access programs (EAPs) (31, 32) or retrospective series (33–35), based on those available data, immunotherapy provides similar intracranial activity in patients with NSCLC, and brain metastases is not a negative prognostic factor. Patients with brain metastases were underrepresented in pivotal immunotherapy trials, accounting for 6.3% to 17.5% of the enrolled patients (22–30, 36, 37). In our analysis, a total of 76 randomized controlled trials were assessed for eligibility, and only 11 (14.5%) trials had a preplanned subgroup analysis according to the brain metastases status (**Figure 1**). The results of our analysis suggested that future immunotherapy trials should ensure a more inclusion of patients with brain metastases, to provide more data on immunotherapy for patients with brain metastases.

A previous meta-analysis examined the efficacy of ICIs in NSCLC patients with different metastatic sites (ie, liver and brain metastases) (38). The results of this study are similar to our analysis, indicating that patients with brain metastases could achieve survival benefits from immunotherapy, compared with conventional therapy. However, in their subgroup analysis, patients with brain metastases could not benefit from ICIs as PD-L1 inhibitors and single agent, which were contrary to our findings. Moreover, their meta-analysis included 8 randomized controlled trials, of which only 3 trials and 259 patients with brain metastases, and did not explore the interaction between the efficacy of immunotherapy and brain metastasis status.

In our subgroup analysis, compared with chemotherapy, a significantly overall survival benefit was only observed for specific patients with brain metastases (ie, immune monotherapy, non-small-cell lung cancer, median follow-up > 20 months, and targeting PD-L1) treated with ICIs. However, due to the small number of patients with brain metastases enrolled in our analysis, it cannot be ruled out that this result was caused by insufficient statistical power.

For subgroups, similar efficacy of immunotherapy in patients with and without brain metastases was observed in the subset of patients (**Figure 3B**). Moreover, the heterogeneity test for brain metastases-related interaction, assessed among each subgroup, was performed. However, only in the subgroup of tumor type, there was significant heterogeneity within patients with NSCLC and SCLC (P = .041), suggesting that the cancer histotype might affect the efficacy of immunotherapy in patients with brain metastases.

There was substantial heterogeneity among studies in both patients with and without brain metastases. It should be noted that when we stratified these studies according to cancer histotype and median follow-up time, the heterogeneity among studies in patients with brain metastases was significantly reduced. which explained the main source of heterogeneity. However, in each subgroup, there was still substantial heterogeneity among studies in patients without brain metastases. It is likely that all these factors, such as therapy type, tumor type, and target of intervention agents, contribute, along with brain metastases, to determine immunotherapy efficacy that differs across studies in the cohort of patients without brain metastases.

The survival of patients with SCLC has not improved until two studies (24, 30) (ie, CASPIAN and Impower133) showing the combination of PD-L1 inhibitors with chemotherapy has survival benefit versus chemotherapy alone. However, such improvement in OS is relatively small, and was not observed in other studies (27, 29) (ie, CA184-156 and KEYNOTE-604). The results of our analysis indicated that SCLC patients with brain metastases could not benefit from the addition of immunotherapy to chemotherapy (HR, 1.10; 95%CI, 0.65-1.55; P = .663), while the significant OS benefit was observed in NSCLC patients (HR, 0.58; 95%CI, 0.41-0.74; P <.001). Based on existing knowledge, we speculated that the result might generate from two biological factors. The first is related to PD-L1 expression in SCLC. Higher PD-L1 expression has shown to be associated with enhanced efficacy of ICIs in NSCLC. Although the clinical meaning of PD-L1 expression remains unclear in SCLC, some studies had demonstrated that the PD-L1-positive rate was relatively low, suggesting the main T-cell co-inhibition pathway may not be the PD-1/L1 axis in SCLC (39–42). The second factor is related to infiltration of tumor-infiltrating lymphocytes (TILs). High TIL burden was observed in brain metastases. Moreover, higher density of TILs in brain metastases were correlated with improved OS. However, a significantly variable and heterogeneous TILs infiltration was reported in tumors of several histotypes, potentially leading to discrepancies in survival among different tumor types (43, 44).

Other clinical or biological factors had been discovered, which could affect the efficacy of immunotherapy in patients with brain metastases, and these factors might influence our results. Clinically, steroids are frequently used in metastatic NSCLC patients, and the use of steroids was associated with a worse outcome (45). In a multicenter cohort study, in multivariate analysis patients with brain metastases with baseline steroids were associated with a poorer survival (HR, 2.37) (46). Biologically, higher PD-L1 expression is a strong predictor of benefit from immune checkpoint inhibitors in NSCLC (41, 42). Recently, a study found the strong correlation of PD-L1 expression between the primary tumor and the brain metastases (47). Meanwhile, Goldberg and colleagues found that higher PD-L1 expression in tumor cells was associated with prolonged OS in NSCLC patients with brain metastases (17).However, as described in our sensitivity analyses, these factors (ie, use of steroids and PD-L1 expression), are not likely to explain our findings.

This meta-analysis also has some limitations. Firstly, we performed this analysis at the trial level, rather than at the individual level. Therefore, we cannot conduct subgroup analysis according to the region (Asia or Europe), disease severity, tumor burden (single or multiple brain metastases) and radiotherapy schemes before immunotherapy, which might affect the efficacy of ICIs. Secondly, it should be noted that the number of patients with brain metastases were relatively low in our analysis. It cannot be ruled out that insufficient statistical power might explain these subgroup analysis results. Thirdly, all of trials in our study only enrolled patients with treated asymptomatic brain metastases; therefore, the extrapolation of the results might be limited.

## Conclusions

Our meta-analysis indicates that lung cancer patients with asymptomatic brain metastases can gain more survival benefit from ICIs, compared with chemotherapy, especially in patients with NSCLC. However, the magnitude of this benefit is brain metastases dependent. We recommend that future immunotherapy trials should ensure a larger inclusion of NSCLC patients with brain metastases, however, more effective immunotherapeutic approaches should be explored in SCLC patients with brain metastases.

## Data Availability Statement

The original contributions presented in the study are included in the article/**Supplementary Material**. Further inquiries can be directed to the corresponding authors.

## Author Contributions

YZ and LM had full access to all of the data in the study and take responsibility for the integrity of the data and the accuracy of the data analysis. YW, QZ, CC, and YH contributed equally to this work and served as co–first authors. Concept and design: YW, YH, YZ, and LM. Acquisition, analysis, or interpretation of data: All authors. Drafting of the manuscript: YW, YH, YZ, and LM. Critical revision of the manuscript for important intellectual content: QZ, CC, and LM. Statistical analysis: YH. Administrative, technical, or material support: YZ and LM. Supervision: YZ and LM. All authors contributed to the article and approved the submitted version.

## Funding

This work was supported by the “Six Talent Peaks” of Jiangsu Provincial Department of Human Resources and Social Security grants 2014-WSN-047; Nanjing Medical Science and Technology Development Key Project grants ZKX15020; and Wu Jieping Foundation grants 320.6750.19081.

## Conflict of Interest

The authors declare that the research was conducted in the absence of any commercial or financial relationships that could be construed as a potential conflict of interest.

## Publisher’s Note

All claims expressed in this article are solely those of the authors and do not necessarily represent those of their affiliated organizations, or those of the publisher, the editors and the reviewers. Any product that may be evaluated in this article, or claim that may be made by its manufacturer, is not guaranteed or endorsed by the publisher.

## Abbreviations

NSCLC, non-small-cell lung cancer; OS, overall survival; ALK, anaplastic lymphoma kinase; EGFR, epidermal growth factor receptor; ICIs, immune checkpoint inhibitors; BBB: blood-brain barrier; ORR, overall response rate; HR, hazard ratio; SCLC, small-cell lung cancer; EAPs, expanded access programs; TILs, tumor-infiltrating lymphocytes.
