# Supplementary material for: Association of Brain Metastases With Immune Checkpoint Inhibitors Efficacy in Advanced Lung Cancer: A Systematic Review and Meta-Analysis
Source: Front Oncol. 2021 Dec 8;11:721760. doi: 10.3389/fonc.2021.721760 (PMC8694212; doi:10.3389/fonc.2021.721760)
Supplement: Supplementary file 1 [file DataSheet_1.docx]

**Supplementary Online Content**

**Supplementary table 1**. Search Strategies for PubMed, Embase and Cochrane Library

**Supplementary table 2**. Risk of Bias

**Supplementary table 3.** Key Inclusion and Exclusion Criteria of Each Trial for Patients with CNS Metastasis

**Supplementary figure 1.** Sensitivity Analysis by Excluding the KEYNOTE-024 Trial. (A) Hazard Ratios comparing overall survival in patients with brain metastases who received immunotherapy vs chemotherapy, excluding the KEYNOTE-024 Trial, (B) The interaction between immunotherapy efficacy and brain metastases, excluding the KEYNOTE-024 Trial

**Supplementary figure 2.** Sensitivity Analysis by Excluding the CheckMate-057 Trial. (A) Hazard Ratios comparing overall survival in patients with brain metastases who received immunotherapy vs chemotherapy, excluding the CheckMate-057 Trial, (B) The interaction between immunotherapy efficacy and brain metastases, excluding the CheckMate-057 Trial

**Supplementary figure 3**. A Funnel Plot of the Interaction between Immunotherapy Effect and Brain Metastases

**Supplementary table 1. Search strategies for PubMed, Embase and Cochrane Library**

| **Database** | **Keywords** |
| --- | --- |
| **PubMed** |  |
| #1 | "Lung Neoplasms"[Mesh] |
| #2 | Neoplasms,Lung[Title/Abstract] OR Lung Neoplasm[Title/Abstract] OR Neoplasm,Lung[Title/Abstract] OR Neoplasms,Pulmonary[Title/Abstract] OR Neoplasm,Pulmonary[Title/Abstract] OR Pulmonary Neoplasm[Title/Abstract] OR Lung Cancer[Title/Abstract] OR Cancer,Lung[Title/Abstract] OR Cancers,Lung[Title/Abstract] OR Lung Cancers[Title/Abstract] OR Pulmonary Cancer[Title/Abstract] OR Cancer,Pulmonary[Title/Abstract] OR Cancers,Pulmonary[Title/Abstract] OR Pulmonary Cancers[Title/Abstract] OR Cancer of the Lung[Title/Abstract] OR Cancer of Lung[Title/Abstract] |
| #3 | #1 OR #12 |
| #4 | immune checkpoint inhibitor[Title/Abstract] OR Programmed Cell Death 1 Receptor[Title/Abstract] OR PD-1 Receptor[Title/Abstract] OR PD 1 Receptor[Title/Abstract] OR Receptor, PD-1[Title/Abstract] OR CD279 Antigen[Title/Abstract] OR Antigen, CD279[Title/Abstract] OR PD1 Receptor[Title/Abstract] OR Receptor, PD1[Title/Abstract] OR Programmed Cell Death 1 Protein[Title/Abstract] OR Antigens, CD279[Title/Abstract] OR CD279 Antigens[Title/Abstract] OR CTLA-4 Antigen[Title/Abstract] OR Antigen, CTLA-4[Title/Abstract] OR CTLA 4 Antigen[Title/Abstract] OR Antigens, CD152[Title/Abstract] OR CD152 Antigens[Title/Abstract] OR Cytotoxic T-Lymphocyte-Associated Antigen 4[Title/Abstract] OR Cytotoxic T Lymphocyte Associated Antigen 4[Title/Abstract] OR Cytotoxic T-Lymphocyte Antigen 4[Title/Abstract] OR Cytotoxic T Lymphocyte Antigen 4[Title/Abstract] OR CD152 Antigen[Title/Abstract] OR Antigen, CD152[Title/Abstract] OR Programmed cell death ligand 1[Title/Abstract] OR PD-L1[Title/Abstract] OR Ipilimumab[Title/Abstract] OR tremelimumab[Title/Abstract] OR pembrolizumab[Title/Abstract] OR nivolumab[Title/Abstract] OR atezolizumab[Title/Abstract] OR durvalumab[Title/Abstract] OR avelumab[Title/Abstract] OR Cemiplimab[Title/Abstract] OR Camrelizumab[Title/Abstract] OR Sintilimab[Title/Abstract] OR Tislelizumab[Title/Abstract] OR Toripalimab[Title/Abstract] |
| #5 | randomized controlled trial[Publication Type] OR controlled clinical trial[Publication Type] OR randomized[Title/Abstract] OR placebo[Title/Abstract] OR drug therapy[MeSH Subheading] OR randomly[Title/Abstract] OR trial[Title/Abstract] OR groups[Title/Abstract] |
| #6 | animals[MeSH Terms] NOT humans[MeSH Terms] |
| #7 | #5 NOT #6 |
| #8 | #3 AND #4 AND #7 |
| **Embase** |  |
| #1 | 'lung cancer'/exp |
| #2 | 'Pulmonary Neoplasms':ab,ti OR 'Neoplasms, Lung':ab,ti OR 'Lung Neoplasm':ab,ti OR 'Neoplasms, Pulmonary':ab,ti OR 'Neoplasm, Pulmonary':ab,ti OR 'Pulmonary Neoplasm':ab,ti OR 'Lung Cancer':ab,ti OR 'Cancer, Lung':ab,ti OR 'Cancers, Lung':ab,ti OR 'Lung Cancers':ab,ti OR 'Pulmonary Cancers':ab,ti OR 'Cancer, Pulmonary':ab,ti OR 'Cancers, Pulmonary':ab,ti OR 'Pulmonary Cancers':ab,ti OR 'Cancer of the Lung':ab,ti OR 'Cancer of Lung':ab,ti |
| #3 | #1 OR #2 |
| #4 | 'immune checkpoint inhibitOR':ab,ti OR 'Programmed Cell Death 1 ReceptOR':ab,ti OR 'PD-1 ReceptOR':ab,ti OR 'PD 1 ReceptOR':ab,ti OR 'ReceptOR, PD-1':ab,ti OR 'CD279 Antigen':ab,ti OR 'Antigen, CD279':ab,ti OR 'PD1 ReceptOR':ab,ti OR 'ReceptOR, PD1':ab,ti OR 'Programmed Cell Death 1 Protein':ab,ti OR 'Antigens, CD279':ab,ti OR 'CD279 Antigens':ab,ti OR 'CTLA-4 Antigen':ab,ti OR 'Antigen, CTLA-4':ab,ti OR 'CTLA 4 Antigen':ab,ti OR 'Antigens, CD152':ab,ti OR 'CD152 Antigens':ab,ti OR 'Cytotoxic T-Lymphocyte-Associated Antigen 4':ab,ti OR 'Cytotoxic T Lymphocyte Associated Antigen 4':ab,ti OR 'Cytotoxic T-Lymphocyte Antigen 4':ab,ti OR 'Cytotoxic T Lymphocyte Antigen 4':ab,ti OR 'CD152 Antigen':ab,ti OR 'Antigen, CD152':ab,ti OR 'Programmed cell death ligand 1':ab,ti OR 'PD-L1':ab,ti OR 'Ipilimumab':ab,ti OR 'tremelimumab':ab,ti OR 'pembrolizumab':ab,ti OR 'nivolumab':ab,ti OR 'atezolizumab':ab,ti OR 'durvalumab':ab,ti OR 'avelumab':ab,ti OR 'Cemiplimab':ab,ti OR 'Camrelizumab':ab,ti OR 'Sintilimab':ab,ti OR 'Tislelizumab':ab,ti OR 'TORipalimab':ab,ti |
| #5 | 'crossover procedure':de OR 'double-blind procedure':de OR 'randomized controlled trial':de OR 'single-blind procedure':de OR random*:de,ab,ti OR factorial*:de,ab,ti OR crossover*:de,ab,ti OR ((cross NEXT/1 over*):de,ab,ti) OR placebo*:de,ab,ti OR ((doubl* NEAR/1 blind*):de,ab,ti) OR ((singl* NEAR/1 blind*):de,ab,ti) OR assign*:de,ab,ti OR allocat*:de,ab,ti OR volunteer*:de,ab,ti |
| #6 | #3 AND #4 AND #5 |
| **Cochrane Library** |  |
| #1 | MeSH: Lung Neoplasms |
| #2 | (Pulmonary Neoplasms):ti,ab,kw OR (Neoplasms, Lung):ti,ab,kw OR (Lung Neoplasm):ti,ab,kw OR (Neoplasms, Pulmonary):ti,ab,kw OR (Neoplasm, Pulmonary):ti,ab,kw OR (Pulmonary Neoplasm):ti,ab,kw OR (Lung Cancer):ti,ab,kw OR (Cancer, Lung):ti,ab,kw OR (Cancers, Lung):ti,ab,kw OR (Lung Cancers):ti,ab,kw OR (Pulmonary Cancer):ti,ab,kw OR (Cancer, Pulmonary):ti,ab,kw OR (Cancers, Pulmonary):ti,ab,kw OR (Pulmonary Cancers):ti,ab,kw OR (Cancer of the Lung):ti,ab,kw OR (Cancer of Lung):ti,ab,kw |
| #3 | #1 OR #2 |
| #4 | (immune checkpoint inhibitor):ti,ab,kw OR (Programmed Cell Death 1 Receptor):ti,ab,kw OR (PD-1 Receptor):ti,ab,kw OR (PD 1 Receptor):ti,ab,kw OR (Receptor, PD-1):ti,ab,kw OR (CD279 Antigen):ti,ab,kw OR (Antigen, CD279):ti,ab,kw OR (PD1 Receptor):ti,ab,kw OR (Receptor, PD1):ti,ab,kw OR (Programmed Cell Death 1 Protein):ti,ab,kw OR (Antigens, CD279):ti,ab,kw OR (CD279 Antigens):ti,ab,kw OR (CTLA-4 Antigen):ti,ab,kw OR (Antigen, CTLA-4):ti,ab,kw OR (CTLA 4 Antigen):ti,ab,kw OR (Antigens, CD152):ti,ab,kw OR (CD152 Antigens):ti,ab,kw OR (Cytotoxic T-Lymphocyte-Associated Antigen 4):ti,ab,kw OR (Cytotoxic T Lymphocyte Associated Antigen 4):ti,ab,kw OR (Cytotoxic T-Lymphocyte Antigen 4):ti,ab,kw OR (Cytotoxic T Lymphocyte Antigen 4):ti,ab,kw OR (CD152 Antigen):ti,ab,kw OR (Antigen, CD152):ti,ab,kw OR (Programmed cell death ligand 1):ti,ab,kw OR (PD-L1):ti,ab,kw OR (Ipilimumab):ti,ab,kw OR (tremelimumab):ti,ab,kw OR (pembrolizumab):ti,ab,kw OR (nivolumab):ti,ab,kw OR (atezolizumab):ti,ab,kw OR (durvalumab):ti,ab,kw OR (avelumab):ti,ab,kw OR (Cemiplimab):ti,ab,kw OR (Camrelizumab):ti,ab,kw OR (Sintilimab):ti,ab,kw OR (Tislelizumab):ti,ab,kw OR (Toripalimab):ti,ab,kw |
| #5 | #3 AND #4 |

**Supplementary table 2. Risk of Bias**

|  | Random Sequence Generation(selection bias) | Allocation concelment(selection bias) | Blinding of participants and personnel(performence bias) | Blinding of outcome assessment(detection bias) | Incomplete outcome data(attrition bias) | Selective reporting(reporting bias) |
| --- | --- | --- | --- | --- | --- | --- |
| Gandhi et al | Low Risk | Low Risk | Low Risk | Low Risk | Low Risk | Low Risk |
| Hellmann et al | Low Risk | Low Risk | At Risk | Low Risk | Low Risk | Low Risk |
| Horn et al | Low Risk | Low Risk | Low Risk | Low Risk | Low Risk | Low Risk |
| Reck et al | Low Risk | Low Risk | At Risk | Low Risk | Low Risk | Low Risk |
| Fehrenbacher et al | Low Risk | At Risk | At Risk | Unclear | Low Risk | Low Risk |
| Reck et al | Low Risk | Low Risk | Low Risk | Low Risk | Low Risk | Low Risk |
| Borghaei et al | Low Risk | Low Risk | At Risk | Low Risk | Low Risk | Low Risk |
| Rudin et al | Low Risk | Low Risk | Low Risk | Low Risk | Low Risk | Low Risk |
| Paz-Ares et al | Low Risk | Low Risk | At Risk | Low Risk | Low Risk | Low Risk |

**Supplementary table 3. Key Inclusion and Exclusion Criteria of Each Trial for Patients with CNS Metastasis**

| **Trial** | **Asymptomatic metastases** | **Active symptomatic metastases/  eptomeningeal disease** | **CNS metastasis  identification** | **Location of**  **CNS metastasis** | **Steroid/anticonvulsant** | **CNS directed therapy** |
| --- | --- | --- | --- | --- | --- | --- |
| KEYNOTE-189 | Eligible | Excluded | CT or MRI | No restriction | off steroids 3 days prior to dosing with study medication | NR |
| CheckMate 227 | Eligible if treated | Excluded | NR | No restriction | NR | NR |
| IMpower133 | Eligible if treated | Excluded | CT or MRI | Only supratentorial and cerebellar metastases allowed | No ongoing requirement for corticosteroids as therapy for CNS disease | Patients with new asymptomatic CNS metastases detected at the screening scan must receive radiation therapy and/or surgery for CNS metastases. |
| KEYNOTE-024 | Eligible if treated | Excluded | NR | No restriction | NR | NR |
| OAK | Eligible if treated | Excluded | CT or MRI | Only supratentorial metastases with no intracranial hemorrhage  were eligible | Anticonvulsants at a stable dose allowed but no ongoing steroids as therapy for CNS disease | No stereotactic radiation within 7 days or whole brain radiation within 14 days prior to study |
| CA184-156 study | Eligible | Excluded | CT or MRI | No restriction | Not requiring treatment with steroids or anticonvulsant medications | If treated with radiation therapy, CNS metastases must be stable with no evidence of progression on scans for at least 30 days from initial radiologic diagnosis of CNS metastases |
| **Trial** | **Asymptomatic metastases** | **Active symptomatic metastases/  eptomeningeal disease** | **CNS metastasis  identification** | **Location of**  **CNS metastasis** | **Steroid/anticonvulsant** | **CNS directed therapy** |
| CheckMate 057 | Eligible if treated | Excluded | CT or MRI | No restriction | stable for at least 2 weeks without the use of steroids or  on stable or decreasing dose of ≤ 10mg daily prednisone (or equivalent) | At least 2 weeks prior to study enrolment |
| KEYNOTE-604 | Eligible if treated | Excluded | CT or MRI | No restriction | Neurologically stable without corticosteroids for ≥ 7 days before starting study treatment | Whole-brain radiation and stereotactic radiosurgery≥14 days before starting study treatment |
| CASPIAN | Eligible if treated | Excluded | CT or MRI | No restriction | Stable off steroids and anticonvulsants for at least 1 month before study entry | Radiation therapy outside of the chest for palliative care is allowed but must be completed before first dose of the study medication |

Abbreviations: CNS, central nervous system; CT, computed tomography; MRI, magnetic resonance imaging; NR, not reported.


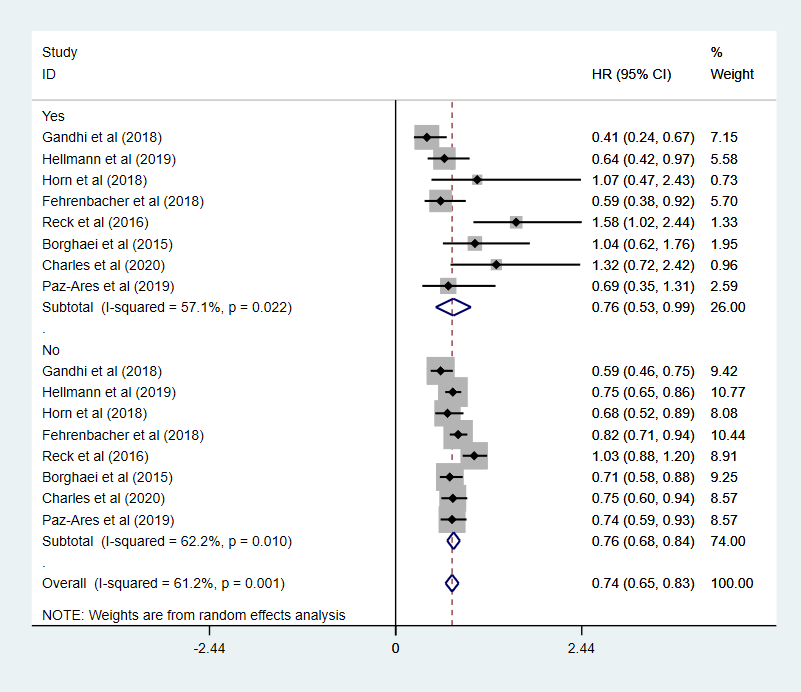


A


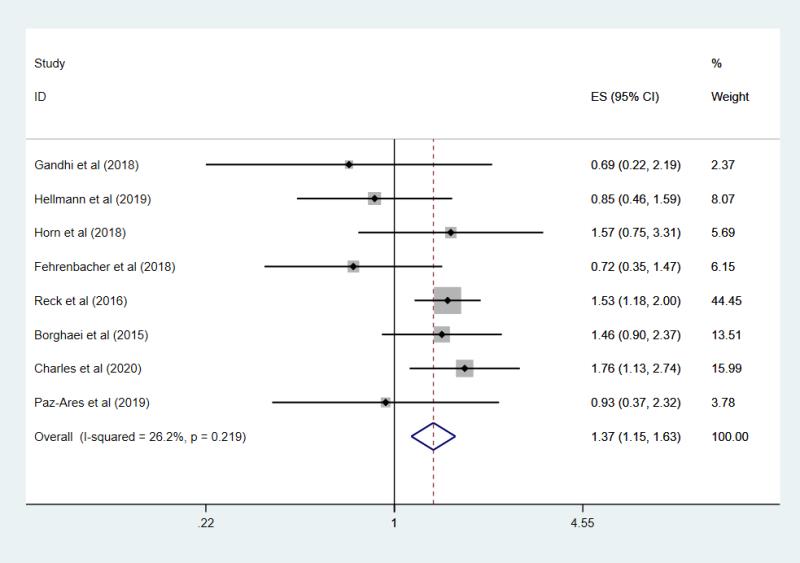


B

**Supplementary figure 1.** Sensitivity Analysis by Excluding the KEYNOTE-024 Trial.


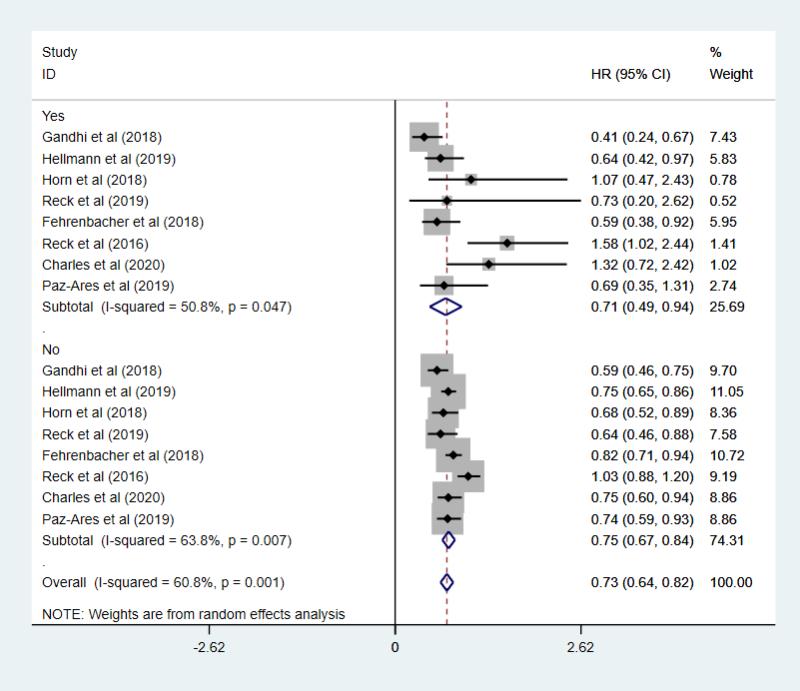


A


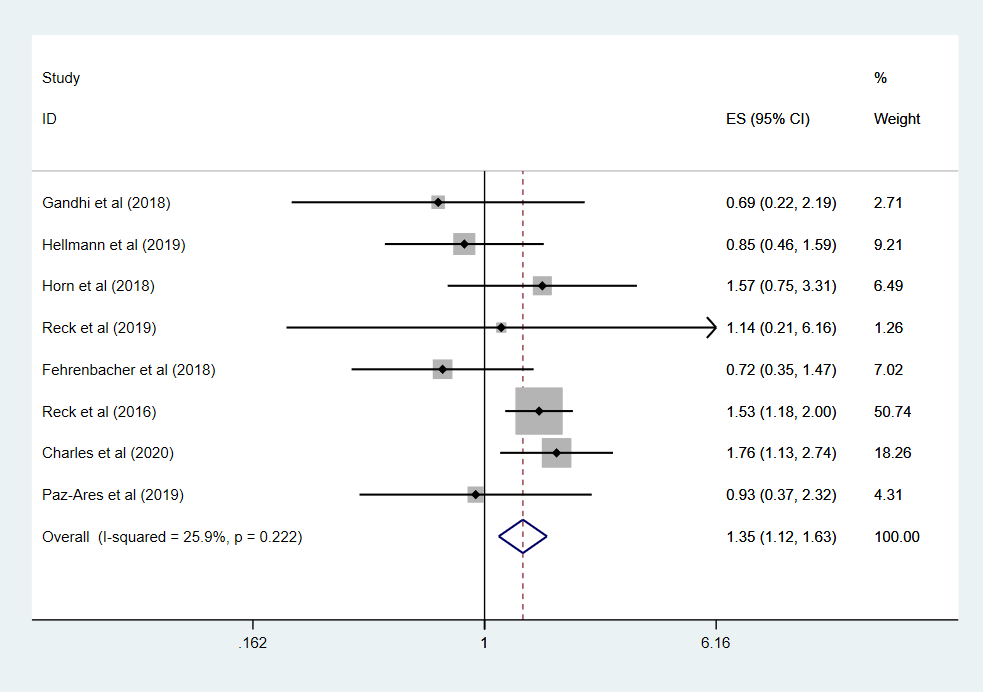


B

**Supplementary figure 2.** Sensitivity Analysis by Excluding the CheckMate-057 Trial.


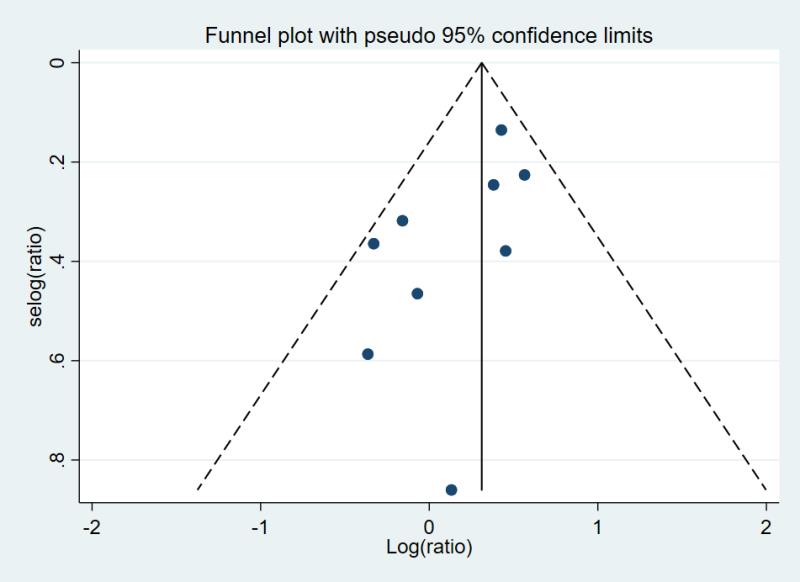


**Supplementary figure 3.** A Funnel Plot of the Interaction between Immunotherapy Effect and Brain Metastases
